# ATP4 and ciliation in the neuroectoderm and endoderm of Xenopus embryos and tadpoles

**DOI:** 10.1016/j.dib.2015.04.003

**Published:** 2015-04-20

**Authors:** Peter Walentek, Cathrin Hagenlocher, Tina Beyer, Christina Müller, Kerstin Feistel, Axel Schweickert, Richard M. Harland, Martin Blum

**Affiliations:** a Institute of Zoology, University of Hohenheim, Garbenstrasse 30, 70593 Stuttgart, Germany; b Department of Molecular and Cell Biology, Center for Integrative Genomics, University of California at Berkeley, Berkeley, California 94720, USA

**Keywords:** Cilia, ATP4a, Gastric H+/K+ATPase, Wnt signaling, Xenopus

## Abstract

During gastrulation and neurulation, *foxj1* expression requires ATP4a-dependent Wnt/β-catenin signaling for ciliation of the gastrocoel roof plate (Walentek et al. *Cell Rep*. 1 (2012) 516–527.) and the mucociliary epidermis (Walentek et al. *Dev. Biol.* (2015)) of *Xenopus laevis* embryos. These data suggested that ATP4a and Wnt/β-catenin signaling regulate *foxj1* throughout *Xenopus* development. Here we analyzed whether *foxj1* expression was also ATP4a-dependent in other ciliated tissues of the developing *Xenopus* embryo and tadpole. We found that in the floor plate of the neural tube ATP4a-dependent canonical Wnt signaling was required for *foxj1* expression, downstream of or in parallel to Hedgehog signaling. In the developing tadpole brain, ATP4-function was a prerequisite for the establishment of cerebrospinal fluid flow. Furthermore, we describe *foxj1* expression and the presence of multiciliated cells in the developing tadpole gastrointestinal tract. Our work argues for a general requirement of ATP4-dependent Wnt/β-catenin signaling for *foxj1* expression and motile ciliogenesis throughout *Xenopus* development.

**Table:** 

**Specification Table**	
Subject area	Biology
More specific subject area	Cell and developmental biology
Type of data	Text file, figures, movies
How data was acquired	Microscopy (fluorescent, confocal, bright-field)
Data format	Analyzed and annotated figures and movies
Experimental factors	NA
Experimental features	Xenopus embryos were manipulated by morpholino oligonucleotide-mediated knockdown and application of pharmacological inhibitors. Gene expression, morphology and cilia function were analyzed by in situ hybridization, immunofluorescence, and quantification of extracellular fluid flow
Data source location	NA
Data accessibility	The data described here is presented in this article in form of figures and supplemental movies

**Value of the data**

• Our results indicate that the ATP4/Wnt/β-catenin module is required for neural *foxj1* expression downstream of, or in parallel to, Hedgehog signaling.
• ATP4 function is required for the generation of cerebrospinal fluid flow.
• *atp4a* and *foxj1* are co-expressed in the gastrointestinal tract.
• The tadpole stomach is lined by multiciliated cells, which generate an extracellular fluid flow.

## 1 Data, experimental design and methods

### 1.1 Analysis of ATP4a/Wnt-dependent *foxj1* expression in floor plate of the neural tube

**Fig. 1:**
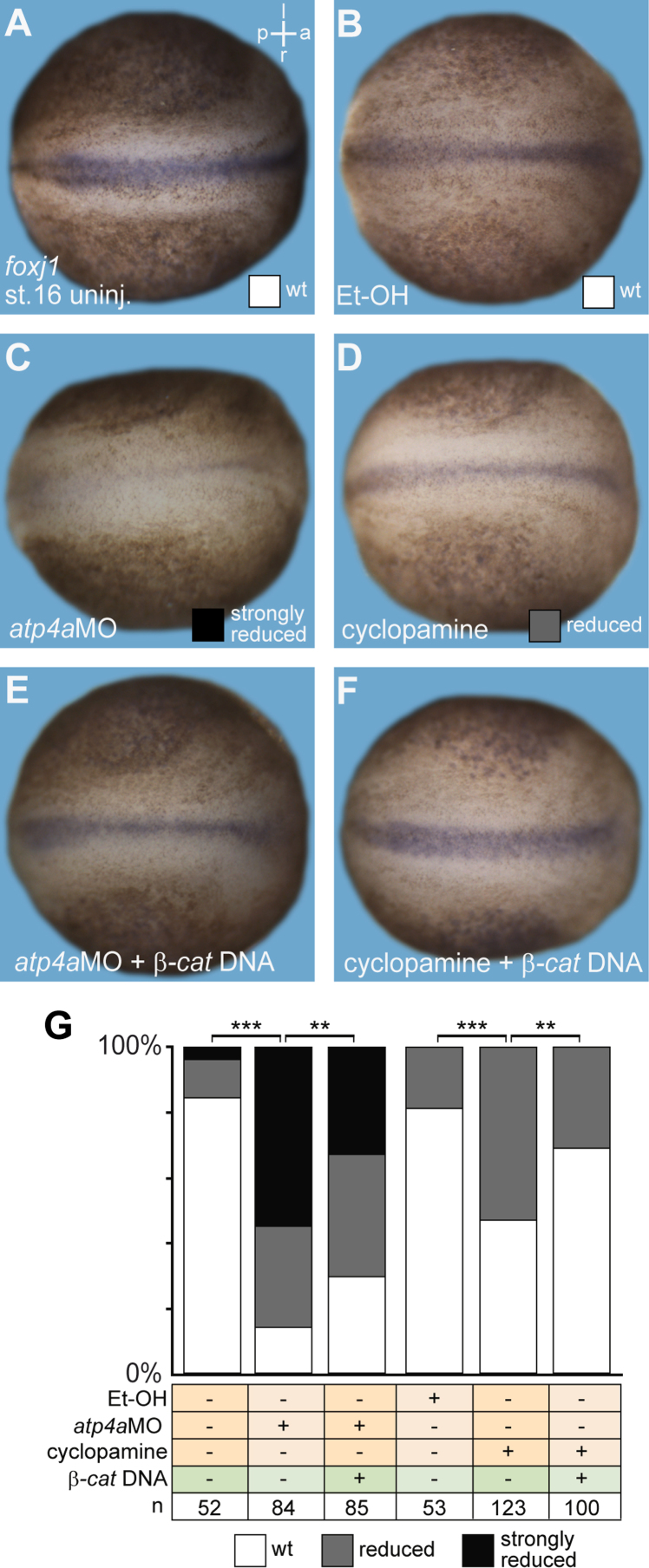
Floor plate *foxj1* expression requires ATP4a and β-catenin downstream or in parallel of Hedgehog signaling. (A–F) WMISH for *foxj1* expression in control and manipulated embryos at stage 16. (A, B) Normal *foxj1* expression in the floor plate of control uninjected (uninj.; A) and ethanol (1%; EtOH; B) treated specimens. (C) Strong reduction of *foxj1* signals in *atp4a* morphants was partially rescued by co-injection of *β-catenin* DNA (*ß-cat.;* E). (D) Inhibition of Hedgehog signaling by cyclopamine treatment decreased *foxj1* expression in the floor plate and was partially rescued by injection of *β-catenin* DNA (*ß-cat.;* F). (G) Quantification of results. a, anterior; l, left; *n*, number of embryos; p, posterior; r, right; st., stage.

The floor plate and the brain represent additional sites of vertebrate *foxj1* expression [1,5,6,13]. We tested whether floor plate expression of *foxj1* required ATP4 and Wnt/β-catenin signaling in *Xenopus* by injection of 1 pmol/injection of *atp4a* morpholino oligonucleotide (*atp4a*MO) targeted to dorso-medial regions of developing embryos. Embryos were injected at the two- to four-cell stage using a Harvard Apparatus or Picospritzer setup in 1× modified Barth׳s solution (MBSH) with 4% Ficoll (BioChemica) and transferred to 0.1× MBSH 15 min after injection. Gene expression was analyzed by whole mount in situ hybridization (WMISH). *atp4a* morphants showed a reduction of *foxj1* expression in the floor plate (*p*<0.001; Fig. 1A,C and G), which was rescued by co-injection of 1 ng/μl *β***-***catenin* DNA (*p*<0.01; Fig. 1E and G).

### 1.2 Monitoring floor plate formation in *atp4a* morphants

**Fig. 2:**
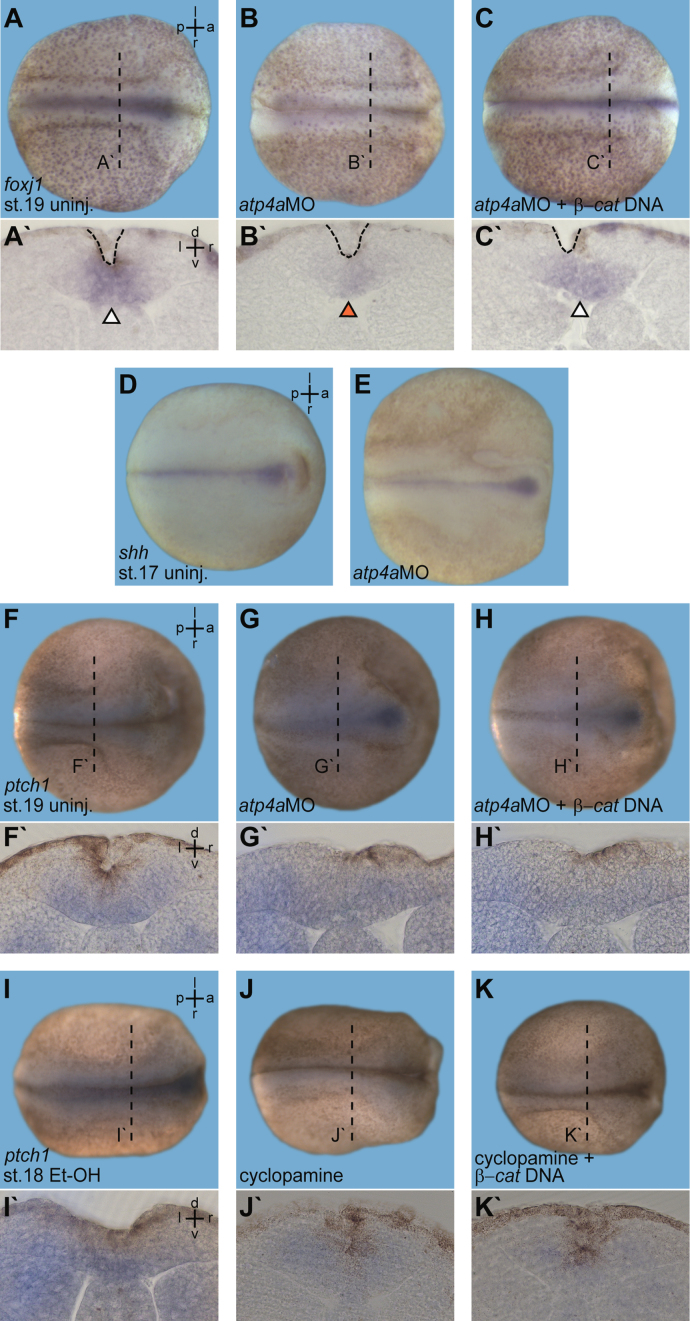
Normal floor plate induction and Hedgehog signaling in ATP4a-deficient embryos. (A–E) Normal floor plate formation in *atp4a* morphants. WMISH for *foxj1* (A–C) and *shh* (D, E) revealed attenuated *foxj1* expression but unaffected floor plate formation, as judged by apically constricted cells (histological vibratome sections in A–C`; planes indicated in A–C) and *shh* expression in *atp4a* morphants. (F–K) Unaffected Hedgehog signaling in *atp4a* morphants with (H) or without (G) co-injection of *ß-catenin* (*β-cat.*) DNA, as judged by WMISH for *ptch1*, a direct Hedgehog signaling target, to monitor activity of Hedgehog signaling, as compared to uninjected controls (uninj.). *ptch1* expression was decreased in cyclopamine-treated embryos (J, K), as compared to ethanol (1%) controls (I), but independent of *β-catenin* DNA injection (K, K`). a, anterior; l, left; p, posterior, right; st., stage.

Formation of the floor plate in *atp4a* morphants was analyzed histologically and by analysis of gene expression. Embryos were embedded in gelatin–albumin and sectioned on a vibratome (30 μm). The floor plate was present, as judged by concentration of pigment due to apical constriction of medial neural plate cells, both in *atp4a* morphants and in specimens co-injected with *β***-***catenin* DNA (Fig. 2A–C, A`–C`). Floor plate-specific *sonic hedgehog* expression (*shh*; [12]) was also present in *atp4a* morphants (Fig. 2D and E).

### 1.3 Analysis of Hedgehog-dependent *foxj1* expression in the floor plate of the neural tube

To analyze if *Xenopus foxj1* expression depended on Hedgehog (HH) signaling, as reported for zebrafish *foxj1* [3,20], embryos were incubated with the HH signaling inhibitor cyclopamine (Selleckchem; solvent: ethanol; concentration: 100 μM). Incubations were performed according to standard procedures [7] from stage 8 until fixation, on embryos treated with Proteinase K (as described; [15]) in order to permeabilize the fertilization membrane. Proteinase K-treated embryos incubated with corresponding concentrations of ethanol were used as controls. Cyclopamine treatment reduced *foxj1* expression in the floor plate (*p*<0.001; Fig. 1B, D and G). Injection of *β***-***catenin* DNA partially rescued floor plate *foxj1* expression in cyclopamine-treated specimens (*p*<0.01; Fig. 1F and G).

### 1.4 Monitoring hedgehog signaling state in *atp4a* morphants and cyclopamine treated embryos

Analysis of *patched 1 (ptch1)* expresson by WMISH was used to monitor HH signaling activity [9]. Embryos were embedded in gelatin–albumin and sectioned on a vibratome (30 μm). *ptch1* expression was downregulated in cyclopamine treated specimens, but unaffected by *atp4a*MO or *β***-***catenin* DNA injection (Fig. 2F–K).

## 2 Analysis of cerebrospinal fluid flow in *atp4a* morphant tadpoles

**Fig. 3:**
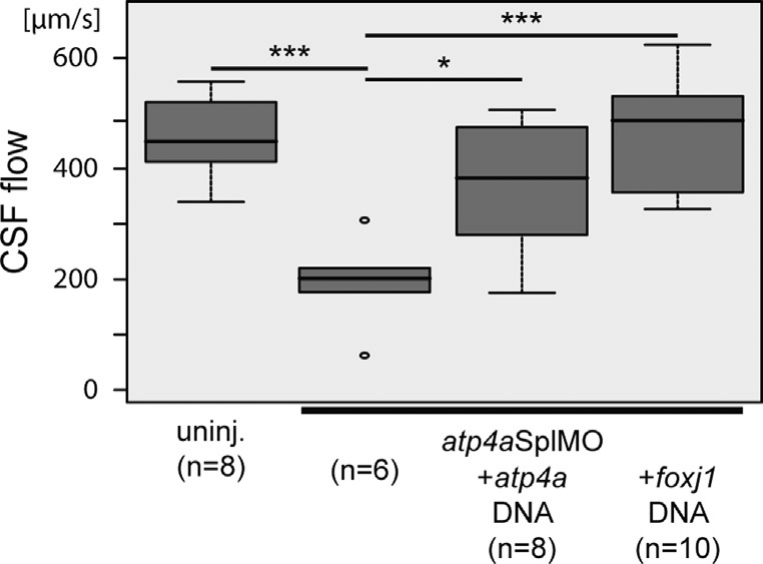
ATP4a is required for *foxj1*-dependent cerebrospinal fluid in the tadpole brain. To investigate whether ATP4 was required for brain cilia, we analyzed cerebrospinal fluid (CSF) flow as a proxy. Injection of fluorescent beads into the brain ventricles at stage 45 revealed a significant reduction of CSF velocity in *atp4a* morphants. In contrast, velocity of CSF flow in *atp4a* morphants was partially rescued by co-injection of either *atp4a* or *foxj1* DNA constructs. cf. Movie 1.

To investigate whether ATP4 was required for brain cilia, we analyzed cerebrospinal fluid (CSF) flow as a proxy. For imaging and calculation of ependymal flow cf. Hagenlocher et al. [6] and Walentek et al. [18]. To facilitate late analysis of *atp4a* morphants, we used 1 pmol/injection of a splice-site MO (*atp4a*SplMO) which targeted the second exon/intron boundary of zygotically expressed pre-mRNA. Injection of fluorescent beads into the brain ventricles at stage 45 revealed a significant reduction of CSF flow velocity in *atp4a* morphants (*p*<0.001; Fig. 3; Movie 1). In contrast, velocity of CSF flow in *atp4a* morphants was increased by co-injection of either *atp4a* or *foxj1* DNA constructs (*p*<0.05/0.001; Fig. 3; Movie 1).

The following is the Supplementary material related to this article Movie 1.

Video 1 ATP4a is required for Foxj1-dependent generation of cerebrospinal fluid flow. Uninjected control (uninj. control) and manipulated embryos were injected with fluorescent beads into the forth brain ventricle of stage 45 tadpoles and cilia-driven fluid flow was recorded for 5.7 sec. Velocity of cilia-driven flow in *atp4a* morphants (*atp4a*SplMO) was strongly reduced and could be restored by co-injection of *atp4a* or *foxj1* DNA. The movie plays at 0.52× real time.

### 2.1 Analysis of *atp4a*SplMO-induced intron retention

**Fig. 4:**
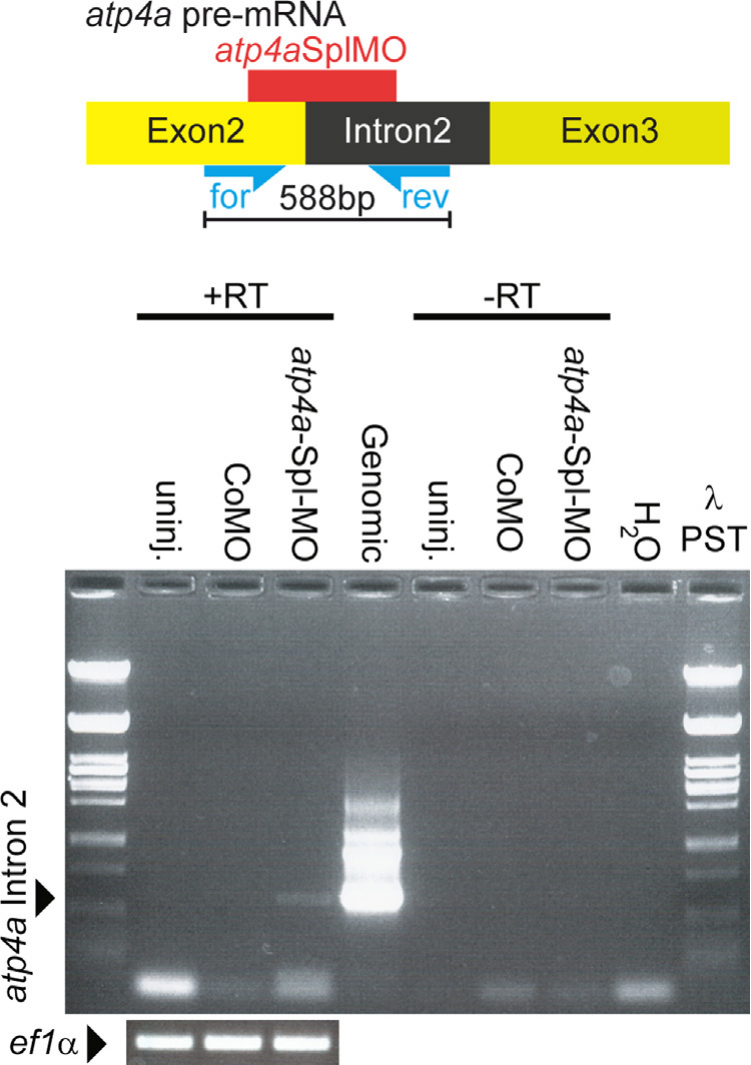
The *atp4*a splice-site MO causes *atp4a* intron 2 retention. To facilitate late analysis of *atp4a* morphants, a splice-site MO (*atp4a*SplMO) was used, which targeted the second exon/intron boundary of zygotically expressed mRNA and caused *atp4a* intron 2 retention. *atp4a*SplMO targeted to gastrointestinal tract caused intron2 retention, as shown by RT-PCR using primers (blue arrows) which bind to exon 2 (yellow) and intron 2 (black). Genomic DNA served as positive control. Total RNA extracts without reverse transcription (-RT) and water (H_2_O) served as negative controls. RT-PCR of *ef1*α served as loading control. λ-phage DNA digested with Pst1 (λ PST) served as size marker.

**Fig. 5:**
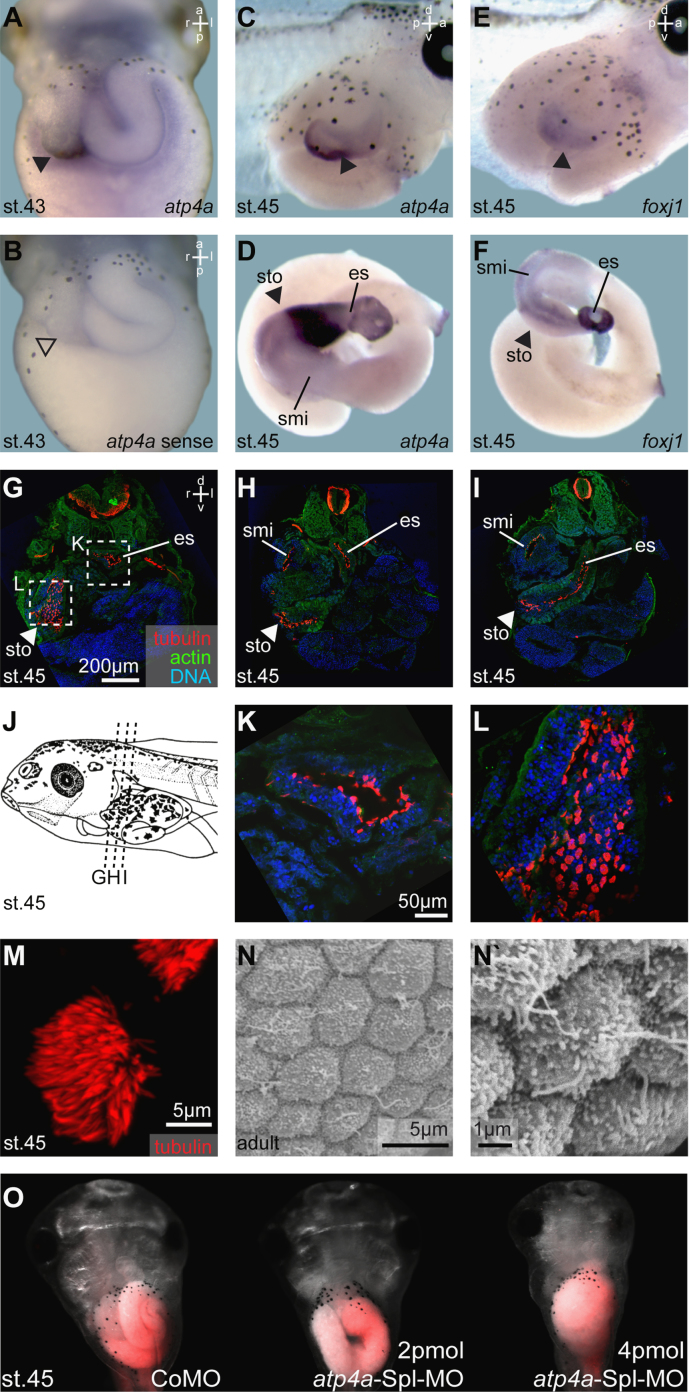
*foxj1* and *atp4a* are co-expressed in the gastrointestinal tract which transiently harbors motile cilia in the stomach. (A–F) WMISH for *atp4a* (A,C,D) and *foxj1* (E,F) in the GI tract of stage 43–45 tadpoles (A–C, E; stomach highlighted by arrowheads; ventral views) and isolated GI tracts (D, F). (B) *atp4a* sense control revealed no staining. Note the co-expression of *atp4a* and *foxj1* at stage 45 (C–F). (G–M) GI tract ciliation as shown by immunofluorescent staining of cilia/tubulin (acetylated-α-tubulin staining, red) and staining for actin (phalloidin, green) as well as nuclei (DAPI, blue) on cryosections from stage 45 tadpoles (planes indicated in J). MCCs (M) were found in the esophagus (es; G–I,K), stomach (sto; G–I,L) and the proximal part of the small intestine (smi; H, I). (N, N`) Scanning electron microscopy analysis of gastric epithelia from adult frogs revealed the presence of short monocilia, but lack of MCCs. (O) Injection of *atp4a*SplMO targeted to the endoderm prevented normal development of the GI tract. Targeting was monitored by co-injection of fluorescent rhodamine dextrane (red). Embryos are shown in ventral view. a, anterior; d, dorsal; es, esophagus; l, left; p, posterior; r, right; st., stage; smi, small intestine; sto, stomach; v, ventral.

4-cell stage embryos were injected four times with 1 pmol/injection of *atp4a*SplMO into the vegetal halve (Fig. 5O), thereby targeting the developing gastrointestinal tract including the stomach, where zygotic *atp4a* expression was previously confirmed [17]. At stage 45 total RNAs were isolated from injected and uninjected tadpoles and *atp4a* intron 2 retention was confirmed (Fig. 4) by standard RT-PCR using the following primers on cDNA and genomic DNA extracts:

*atp4a*Ex2-for 5′-GCATGAAAAAATGGAC-3′;
*atp4a*Int2-rev 5′-TCCTGTCTGCCAATAAACCC-3′;

RT-PCR for *elongation factor 1α* (*ef1a*) was used as loading control employing the following primers:

forward 5′-CAGATTGGTGCTGGATATGC-3′;
reverse 5′-ACTGCCTTGATGACTCCTAG-3′.

## 3 Analysis of *atp4a* and *foxj1* expression in the gastrointestinal tract

High levels of *atp4a* transcripts were found in the stomach of the tadpole (Fig. 5A and C), where ATP4 localization and function have been previously described [8]. We also observed weaker *atp4a* expression in the embryonic esophagus and the proximal small intestine (Fig. 5D). Analysis of *foxj1* mRNA transcription in stage 45 tadpoles revealed expression in the very same regions of the gastrointestinal (GI) tract (Fig. 5E and F).

### 3.1 Analysis of multiciliated cells in the gastrointestinal tract

Gastrointestinal (GI) tract cilia have previously only been reported in the esophagus of *Xenopus* tadpoles [4]. In order to test whether other parts of the GI tract were ciliated, we stained for acetylated-α-tubulin by immuno-histochemistry (Fig. 5G–L). GI tract ciliation was analyzed on cryosections (40 μm) of embryos embedded in O.C.T. (Tissue-Tek) using standard procedures. This analysis confirmed the presence of multiciliated cells (MCCs) in the esophagus and identified the stomach and the proximal small intestine as additional sites of ciliated cells in tadpoles (Fig. 5G–M). In contrast to tadpoles, only short monocilia of about 2 μm length were detected in the adult gastric epithelium by scanning electron microscopy (Fig. 3N and N`).

### 3.2 Assessment of extracellular fluid flow in the gastrointestinal tract

In order to assess motility of endodermal MCCs, the anterior portion of the GI tract of anesthetized stage 45 tadpoles was dissected and incubated in 0.1× MBSH containing benzocaine (Sigma) to prevent peristaltic movements. FITC-conjugated latex beads (FluoSpheres^®^ carboxylate-modified microspheres, 0.5 μm, yellow–green fluorescence (505/515), 2% solids, Life Technologies; diluted to 0.04% in 0.1× MBSH) were diluted to 0.04% in 0.1× MBSH and applied to the anterior esophagus using a Harvard Apparatus injector. The chamber was sealed and imaged for 2 min using epifluorescent illumination at 10× magnification on a Zeiss Axioskop 2 microscope (Movie 2). Beads were transported through the esophagus and stomach until they reached the small intestine, i.e. the region where ciliation starts to decline.

The following is the Supplementary material related to this article Movie 2.

Video 2 Gastrointestinal multiciliated cells drive anterior to posterior fluid flow. The anterior GI tract was freshly prepared from anesthetized stage 45 tadpoles and fluorescent beads were injected into the esophagus (es). Movement of beads was recorded for 2 min. A bright-field picture of the tissue is displayed on the left side. Anatomical parts of the GI tract are as annotated: esophagus (es), stomach (sto) and small intestine (smi). Beads injected into the opening of the esophagus were transported posteriorly. Movement of beads stopped at the transition to the small intestine. The movie plays at 10.9× real time.

### 3.3 ATP4a loss of function in the gastrointestinal tract

To test the functional relevance of ATP4a in the context of GI tract ciliation, *atp4a*SplMO was injected vegetally to target the endoderm (Fig. 5O). Although inhibition of endodermal ATP4a function reduced intestinal *foxj1* expression (not shown), it also interfered with normal development of the GI tract (Fig. 5O), a phenotype reminiscent of failure in Wnt-dependent specification of the proximal GI tract [21].

## 4 Ethics statement

All animals were treated according to the German regulations and laws for care and handling of research animals, and experimental manipulations according to §6, article 1, sentence 2, no. 4 of the animal protection act were approved by the Regional Government Stuttgart, Germany (Vorhaben A 365/10 ZO “Molekulare Embryologie”).

This work was also done with approval of University of California, Berkeley׳s Animal Care and Use Committee. University of California, Berkeley׳s assurance number is A3084-01, and is on file at the National Institutes of Health Office of Laboratory Animal Welfare.

### 4.1 Statistical evaluation of results

Statistical evaluation of experiments represented by bar graphs was performed using chi-square tests (http://www.physics.csbsju.edu/stats/contingency.html). Statistics of experiments represented by box plots were calculated by Wilcoxon sum of ranks (Mann–Whitney) tests (http://www.fon.hum.uva.nl/Service/Statistics/Wilcoxon_Test.html).

### 4.2 Constructs used for Manipulation of embryos

Morpholino oligonucleotides (MOs) were obtained from Gene Tools:

– *atp4a*MO (5′-GTCATATTGTTCCTTTTTCCCCATC-3′) 1 pmol,
– *atp4aSpl*MO (5′-CCCCCCCCCCCATTTCTTACAATGT-3′) 1 pmol.

The following DNA constructs were used for injections after purification using a PureYield Plasmid Midiprep kit (Promega):

– *atp4a*-CS2^+^MT [17] 1 ng/μl,
– *foxj1*-CS2^+^ [16] 0.5 ng/μl,
– *β-catenin*-*gfp*-CS2^+^ [10] 1 ng/μl.

Drop size was calibrated to about 7–8 nl per injection. Rhodamine-B or Cascade-blue dextran (0.5–1.0 mg/ml; Molecular Probes) were co-injected and used as lineage tracer.

### 4.3 Whole-mount in situ hybridization

Embryos were fixed in MEMFA for 1–2 h and processed following standard protocols. Digoxigenin-labeled (Roche) RNA probes (*atp4a* and *foxj1*, [17]; *shh* probe was generated according to NM_001088313; *ptch1*, [11]) were prepared from linearized plasmids using SP6, T3, or T7 RNA polymerase (Promega). In situ hybridization was conducted following standard procedures.

### 4.4 Immuno-histochemistry and scanning electron microscopy

Immuno-histochemistry followed standard protocols, using antibodies specific for acetylated-α-tubulin (mouse, 1:700; Sigma), anti-mouse Cy3 (sheep, 1:250; Sigma); anti-rabbit Alexa-555 (1:250; Invitrogen), anti-mouse Alexa-555 (1:250; Invitrogen). Cell boundaries were visualized by Alexa 488-conjugated phalloidin (Invitrogen), which stained the actin cytoskeleton. DAPI (Invitrogen) was used to visualize nuclei. Imaging was performed on a Zeiss LSM710. Maximum intensity projections of confocal z-scans were computed using ImageJ [14]. Scanning electron microscopy was as previously described [2].

## Funding

Work in the Blum lab was supported by a grant from the Deutsche Forschungsgemeinschaft (BL-285/9) to MB. *Xenopus* work in the Harland lab was supported by the National Institutes of Health (NIH) (GM42341).

PW and TB were recipients of Ph.D. fellowships from the Landesgraduiertenförderung Baden-Württemberg. PW and open-access publication of this article were supported by a postdoctoral fellowship form the Deutsche Forschungsgemeinschaft (Wa 3365/1-1). CH and KF are indebted to the Baden-Württemberg Stiftung for the financial support of their research by the Eliteprogramme for Postdocs. KF was supported by a Margarete-von-Wrangell fellowship, funded by the European Social Fund and by the Ministry Of Science, Research and the Arts in Baden-Württemberg.

## Authors contribution

PW initiated, conducted and analyzed experiments. TB performed electron microscopy. CH and KF helped significantly with ependymal flow. CM contributed to GI analysis. PW, AS and MB planned experiments and interpreted the results. RMH helped with interpretation of data and writing of the manuscript. PW and MB wrote the manuscript with input from all authors.
